# Community, State, and Federal Approaches to Cumulative Risk Assessment: Challenges and Opportunities for Integration

**DOI:** 10.3390/ijerph120504546

**Published:** 2015-04-24

**Authors:** Timothy M. Barzyk, Sacoby Wilson, Anthony Wilson

**Affiliations:** 1 National Exposure Research Laboratory, U.S. Environmental Protection Agency, Research Triangle Park, NC 27709, USA; 2 School of Public Health, University of Maryland, College Park, MD 20740, USA; E-Mail: swilson2@umd.edu; 3 Oak Ridge Institute for Science and Education (ORISE), U.S. Environmental Protection Agency, Research Triangle Park, NC 27709, USA; E-Mail: wilson.anthony@epa.gov

**Keywords:** cumulative risk assessment, environmental health, community-based participatory research, exposure assessment, susceptibility, vulnerability

## Abstract

Community, state, and federal approaches to conventional and cumulative risk assessment (CRA) were described and compared to assess similarities and differences, and develop recommendations for a consistent CRA approach, acceptable across each level as a rigorous scientific methodology, including partnership formation and solution development as necessary practices. Community, state, and federal examples were described and then summarized based on their adherence to CRA principles of: (1) planning, scoping, and problem formulation; (2) risk analysis and ranking, and (3) risk characterization, interpretation, and management. While each application shared the common goal of protecting human health and the environment, they adopted different approaches to achieve this. For a specific project-level analysis of a particular place or instance, this may be acceptable, but to ensure long-term applicability and transferability to other projects, recommendations for developing a consistent approach to CRA are provided. This approach would draw from best practices, risk assessment and decision analysis sciences, and historical lessons learned to provide results in an understandable and accepted manner by all entities. This approach is intended to provide a common ground around which to develop CRA methods and approaches that can be followed at all levels.

## 1. Introduction

Cumulative risk assessment (CRA) is defined by the United States Environmental Protection Agency (EPA) as an analysis, characterization, and possible quantification of the combined risks to health or the environment from multiple agents or stressors [1]. CRA is also a tool for organizing and analyzing information to examine, characterize, and possibly quantify the combined adverse effect on human health or ecologic resources from multiple environmental stressors [2]. To date, both within and outside the EPA, CRA has been a conceptual framework that includes consideration of multiple stressors, but other factors as well, such as stakeholder participation, non-chemical stressors, the role of susceptibility and vulnerability on impacts, and development of risk management options. This approach is intended to produce an overall assessment of human and/or ecological health backed by scientific rigor, but cognizant of social, economic, and other real-world considerations; many of these aspects are not covered by conventional risk assessment. In practice, CRA has been fragmented depending on the needs of the project and purview of the lead investigators, and no standardized method has been adopted or recognized.

This paper examines a variety of risk assessment approaches at community, state, and federal levels in order to compare and contrast their adoption of CRA principles—even if they were not originally intended as CRAs—in order to highlight advantages and limitations to CRA, and to develop recommendations for a consistent and generally agreed-upon methodology. Two important aspects of CRA are the risk analysis (*i.e.*, risk ranking) and the risk management decisions that come from it. CRA risk analysis needs to be able to compare disparate stressors and account for expert values, and risk management need to reflect the feasibility of addressing multiple stressors in the context of available resources and stakeholder needs.

Often, different imperatives of the key actors in a CRA, which could represent a broad group of individuals, organizations, or agencies, compromise the effectiveness of assessments and resultant management strategies. However, CRA is intended to use this diversity to its advantage, so it is possible that the lack of a consistent and agreed-upon approach or methodologies is compromising this potential benefit of a broad partnership. Communities want CRA to more closely reflect their exposure realities and take into consideration the potential costs to their health, quality of life, and economic well-being. States must consider the transparency of their scientific methods and subsequent allocation of resources to affected communities [3]. Federal approaches should be unbiased and transferable across a range of potential scenarios [1,2,4]. To meet the complex challenges of the new millennium, it has been argued that decision-makers should concentrate on a variety of assessment-related strategies; for example, cooperative and voluntary approaches, green design, sustainability, holistic multimedia approaches, place-based environmental decisions, flexible and easy-to-adjust rules, and outcome-based standards [2,4,5,6,7]. A consistent CRA methodology that appeals to multiple actors would help to achieve this.

This study provides an overview of community, state, and federal risk assessment approaches with special emphasis on the adoption (or lack of) of CRA principles. These approaches are often highly tailored to their particular application; even though their goals might be similar (to assess and reduce risk), the approaches generally are not. Without a consistent approach, it is questionable whether a project will be valued beyond its immediate audience, and new projects will have to continue to develop their own approaches. While each assessment may be unique based on the stressors and populations of interest, a consistent approach would ensure that results can be shared across community, state, and federal levels based on rigorous science and achievable goals.

## 2. Background

CRA represents a procedural method that addresses the challenge of real-world scenarios involving multiple stressors, actors, impacts, and solutions. It goes beyond single-chemical risk assessment, a simple characterization or description of issues, or determination of toxicological endpoints of chemical mixtures. CRA promotes use of the analytic-deliberative process wherein experts, stakeholders (e.g., impacted individuals), and policy makers engage early and throughout the assessment [1]. Ideally, it accounts for social, environmental, and economic considerations to promote long-term sustainability of solutions. As such, a CRA can be a dynamic process of personal engagement, risk analysis, characterization, and management. Some of the most important aspects of CRA are outlined below.

### 2.1. Cumulative versus Conventional Risk Assessment

**Figure 1:**
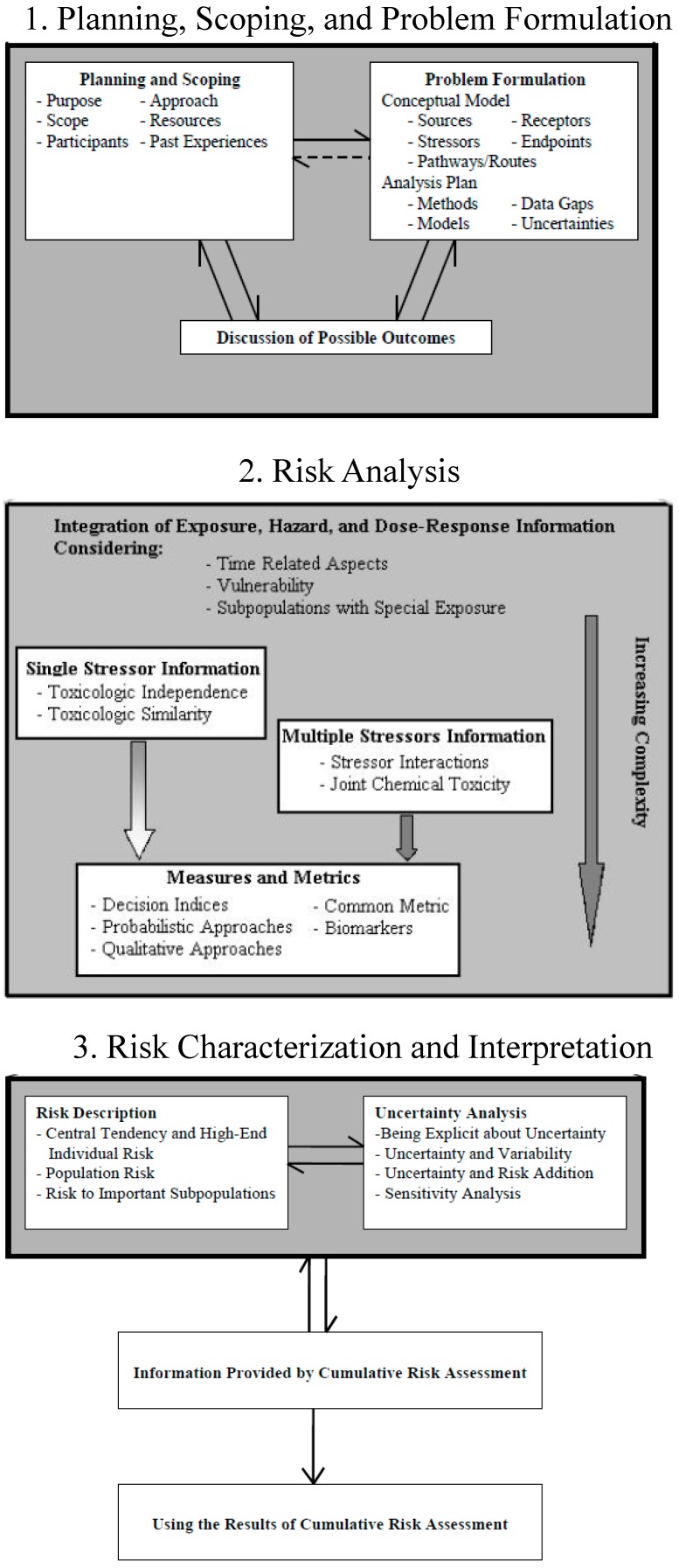
Three phases of a cumulative risk assessment highlighting several features of each; from the EPA *Framework for Cumulative Risk Assessment* (2003) [1].

The four steps to a conventional human health risk assessment (RA) are: (1) hazard identification, (2) dose-response assessment, (3) exposure assessment, and (4) risk characterization (http://www.epa.gov/risk_assessment/health-risk.htm). In contrast, the EPA describes three phases to a CRA [1]: (1) the planning, scoping, and problem formulation phase, (2) the analysis phase, and (3) the risk characterization and interpretation phase (Figure 1). The CRA analysis phase closely reflects conventional RA, except that it includes consideration of synergistic or antagonistic stressor interactions, susceptibility and vulnerability, and chemical and non-chemical stressors; this phase seeks to quantify risk from multiple stressors.

Phases 1 and 3 of CRA expand its scope beyond conventional RA in several ways, calling for meaningful risk communication and the development of risk management options. Risk communication is the process of informing stakeholders about the environmental health risks in a transparent and understandable manner. It represents an ongoing and inclusive dialogue between experts, decision-makers, and stakeholders, the timing of which varies with the situation and complexity of the analysis. The goal of risk communication is to increase community involvement in the decision-making process and environmental remediation efforts, to increase the risk assessor’s awareness of what the community perceives as risks, and to promote understanding of how regulations and policies are related to risk assessment and decision-making (e.g., explaining the limits of federal policies in addressing risks as compared to local, community-based efforts) [8].

Risk management is the process that determines whether or how much to reduce risk through some action, typically related to site remediation and the removal of a stressor like contaminated soil, or the use of filters for contaminated water. Risk management is not considered an integral part of a conventional RA, typically occurring after risk characterization as its own procedure. However, in CRA, risk management should be considered during not only the risk analysis phase, wherein decisions are made based on the information collected during the analysis phase, but also early in the assessment process during planning, scoping and problem formulation. Consideration of potential options for risk reduction provide context and bounds on what can potentially be done. While this in no way should influence the risk analysis itself, it does promote understanding between experts, decision-makers, and stakeholders (*i.e.*, the public or affected individuals) as to the objectives of the CRA and the potential solutions. Also, in addition to pollutant-reduction actions, CRA risk management options may include development or implementation of policies, outreach and education about exposure reduction actions, or additional research on the CRA issues. For example, many researchers use community engagement approaches, including the community-based participatory research (CBPR) framework, to involve key stakeholders in all aspects of the research [9,10,11,12,13]. Four ways that CRA differs from conventional RA include, (1) CRA risk analysis does not necessarily have to deliver an absolute and quantitative estimate of health risk [1,2,8]. Indicators or surrogates that represent a health risk (e.g., proximity to pollution sources), or qualitative relations (e.g., anecdotes of health impacts) may be more appropriate depending on the data required to understand risks and exposures better [1,2,8]; (2) combined effects of more than one agent or stressor are assessed; (3) attention is shifted from a chemical focus (*i.e.*, source-to-exposure pathway) to a population-based assessment of individuals or communities and the multiple stressors to which they are exposed [1,2,8]; (4) evaluation of cumulative risk broadens the spectrum of environmental stressors being assessed beyond the traditional, nearly exclusive focus on chemicals [2].

### 2.2. Multiple-Risk Quantification and Decision Analysis

Risks can generally be defined as the product of the probability of a hazardous event occurring and the adverse consequences that result due to its occurrence; in general terms, these have been described as the likelihood and consequence of an event. Exposure is both a function of actual contact with a stressor as well as the magnitude, concentration (or strength), duration, and possibly spatial extent of the exposure. In addition to exposure, affected individuals may have a greater likelihood or magnitude of exposure, or be more sensitive and thus more susceptible to adverse effects (greater consequence); these populations deserve greater consideration than others and hence greater weighting of risks. Toxicity and exposure values can be used to estimate absolute measures of risk, but semi-quantitative methods that use indicators or surrogates can also be used (e.g., proximity to pollution sources, total emissions per unit area, or number of affected individuals).

In addition to risk quantification methods such as dose-addition or grouping chemicals by a common mode of action (MOA), successful CRAs include a combination of assessment and dialogue, such as that reflected in the “analytic-deliberative” approach [14]. This approach incorporates the best available knowledge with listening and communication skills, and the ability to articulate, evaluate, and refute arguments about an issue [14]. It includes affected individuals, topical experts, and policy makers in the assessment and decision-making process.

Decision analysis methods include the ability to analyze risk perceptions and include expert and stakeholder values in the decision-making process [15,16]. These methods help to address a great deal of variance when lay persons are asked to give their best risk estimate [15,16]. Significant community involvement helps to determine the social, economic and cultural parameters of any CRA [17] and in selecting and implementing appropriate risk management strategies that are culturally sensitive, locally relevant, and community-driven to reduce exposure, eliminate risk, and improve environmental and public health.

### 2.3. Environmental Justice

Environmental justice (EJ) community residents live in or are exposed to high concentrations of multiple chemical, biological, and physical agents as well as other nonchemical stressors, including social determinants of health such as violence, crime, social disorder, racism, discrimination, socioeconomic status (SES), and poverty. Past and current risk assessments have neglected to account for multiple and cumulative exposures in vulnerable populations and in communities with the highest burden of environmental hazards that are maximally exposed to environmental contamination and nonchemical stressors, including psychosocial stressors. [17]. The National Environmental Justice Advisory Council (NEJAC) advocated a “bias for action,” emphasizing early recognition of potential risks and intervention planning even while more-refined assessments are proceeding [18].

## 3. Methods

This section describes community, state, and federal approaches to risk assessment and their overlap with CRA principles. The selection criteria for the community, state, and federal examples are described under their respective sections. Results of this overview were then summarized according to the three CRA phases: (1) planning, scoping, and problem formulation, (2) risk analysis, and (3) risk characterization and interpretation. The Discussion covers the challenges of conducting a CRA, and provides recommendations for developing a consistent approach.

### 3.1. Community

Community examples were chosen from a literature search and include eight studies related to multi-stressor quantification and three with significant stakeholder involvement and engagement. The following two sections highlight studies with a strong focus on quantification and engagement, respectively. This combination is intended to capture aspects of analytical approaches, stakeholder engagement, and risk management practices.

#### 3.1.1. Stressor Quantification

Eight projects developed methods to quantify impacts from a range of stressors; most of these included some level of stakeholder involvement or community-based research. Table 1 presents a summary of these projects, including the problems addressed, study designs, and primary findings.

**Table 1:** Overview of community based projects involving quantification of cumulative risk.

Study & Topics	Purpose or Problem	Study Design	Primary Findings
Sadd *et al*., (2011) [3] Air and Social Environment	Development of the Environmental Justice Screening Method (EJSM) to Examined the relative rank of cumulative impacts and social vulnerability within metropolitan regions.	EJSM uses 23 health, environmental and social vulnerability measures organized along three categories: (1) hazard proximity and land use; (2) estimated air pollution exposure and health risk; and (3) social and health vulnerability in the Los Angeles area.	Areas with high hazard proximity and sensitive land use scores correspond to Areas with high hazard proximity and sensitive land use scores corresponded with dense populations and major industrial centers or transportation corridors. Health risk and exposure scores had little fine-scale variation and broad areas with a single score. Cumulative impact (CI) scores were normally distributed, with highest scores corresponding to communities near ports and airports.
Clougherty *et al*., (2007) [19] Air, Social Environment, and Health Impacts	Examined the role of exposure to violence (ETV), a chronic stressor, in altering susceptibility to traffic-related air pollution in asthma etiology.	GIS-based models estimated residential exposures to traffic-related pollution for 413 children in East Boston, MA, between 1987 and 1993, using monthly NO_2_ measurements for 13 sites over 18 years. Pollution estimates were merged with questionnaire data on lifetime exposure to violence, and effects of both on childhood asthma etiology were examined.	Found elevated risk of asthma with a one standard deviation (4.3 ppb) increase in NO_2_ exposure among children with above-median ETV (odds ratio = 1.63; 95% confidence interval = 1.14–2.33). Demonstrated an association between traffic-related air pollution and asthma solely among urban children exposed to violence.
Clougherty and Kubzansky (2009) [20] Health Impacts and Social Environment	Synthesized relevant research from social and environmental epidemiology, toxicology, immunology, and exposure assessment to provide a framework for environmental health researchers aiming to investigate health effects of environmental pollution combined with social or psychological factors.	Reviewed existing epidemiologic and toxicological evidence on synergistic effects of stress and pollution.	Described Physiologic effects of stress. Addressed key issues related to measuring and evaluating stress as it relates to physical environmental exposures and sus­ceptibility.
Brody *et al*., (2009) [21] Air and Health Impacts	Tested for chemical markers of oil refinery emissions in homes; characterized cumulative effects of emissions in an EJ community by measuring a large and diverse set of pollutants from outdoor and indoor sources; assessed geographic and sociodemographic differences in endocrine disrupting compound (EDC) exposures.	The investigators analyzed indoor and outdoor air from 40 homes in industrial Richmond, CA, and 10 in rural Bolinas, CA, for 153 compounds, including particulates and endocrine disruptors.	Detected eighty outdoor compounds in Richmond and 60 in Bolinas; Richmond concentrations were generally higher, due to heavy oil combustion from oil refining and shipping. Paired outdoor-indoor measurements were correlated to industry- and traffic-related pollutants. Indoor air quality is an important indicator of the cumulative impact of outdoor emissions in fence-line communities.
Morello-Frosch and Shenassa (2006) [22] Psychosocial Stressors and Environmental Hazards	Presented evidence that individual-level and place-based psychosocial stressors may combine with environmental pollutants and have adverse health effects, explaining maternal and child health (MCH) disparities.	Proposed a conceptual framework for holistic approaches to future MCH research that elucidates the interplay of psychosocial stressors and environmental hazards to better explain drivers of MCH disparities.	Suggested that a holistic approach to future MCH research that seeks to untangle the double jeopardy of chronic stressors and environmental hazard exposures could help elucidate how the interplay of these factors shapes persistent racial and economic disparities in MCH.
Su *et al*., (2009) [23] Air and Social Environment	Proposes an index to assess cumulative environmental hazard inequalities in socially disadvantaged groups and neighborhoods in the Los Angeles region of California.	Extended the concentration index to summarize inequality in the distribution of multiple pollutants across socioeconomic and racial/ethnic groups. Index used population ranked by area-based racial, ethnic or socioeconomic composition, and the cumulative environmental hazard, aggregated with various weighting functions.	Analyzed single and cumulative environmental inequalities in exposure to NO_2_, PM_2.5_ and diesel PM; cancer risk; poverty measures; and racial/ethnic population composition. Environmental inequality curves were significantly different from the equality line. Demonstrated that environmental inequalities exist for non-white populations as well as for poorer populations in Los Angeles.
Fox *et al*., (2002) [24] Health Impacts	Advanced CRA methods and tested their application in a community case study. Cumulative risk and health assessments were compared for south and southwest Philadelphia communities.	Obtained mortality data by from the city of Philadelphia, using deaths for 1990 ( *n* = 3151) and for 1988–1992 (*n* = 16,168). Used air pollutant data for all census tracts as a proxy for human exposure. Conducted cumulative risk scoring using two toxicological databases, a multi-end point toxicological database and the EPA Cumulative Exposure Toxicity Database (CETDB).	Analysis found correlations between cumulative risk and mortality measurements for whites and non-whites when risk when using the multi-end point toxicological database. Statistically significant increases in total and respiratory mortality were associated with increases in cumulative risk scores. Regression analyses that controlled for percent non-white population and per capita income indicated that environmental effects on health were independent of race and income.
Krieg and Faber (2004) [25] Toxic Sites	The EJ literature is characterized by a failure to measure overall impact from an extensive range of ecological hazards effectively. Limitations on available data make this a serious problem for present and future studies.	Developed and implemented a cumulative measure of negative environmental impacts by controlling for the density and severity of ecological hazardous sites and facilities within every community in the state.	Found that exposure patterns take a generally linear distribution when analyzed by race and class. Findings suggest that environmental injustice existed on a consistent continuum for nearly all communities.

#### 3.1.2. Stakeholder Engagement

In the early 1990s, citizens from Chester, Pennsylvania, a classic EJ community, requested that a cumulative risk study be performed for the multiple air pollution sources in the community [26]. The EPA conducted an evaluation that included a multiroute chemical risk assessment and a survey of health outcomes in the city [27,28]. This was the first citizen-driven EPA CRA to incorporate community health data into a study to “more accurately address community concerns, and more appropriately characterize and assess the potential risk and exposure of the residents” [26]. Information about cancer disparities, elevated lead levels, exposure disparities, and underlying vulnerabilities was communicated to risk managers [26,28].The information helped the City of Chester obtain funding for its childhood lead poisoning program; monies from the CDC for health outreach; funding for an inspector to review physical stressor issues (odor and noise); resource mobilization from local businesses; and assistance from AmeriCorps VISTA (Volunteers in Service to America) to clean up refuse in the city [26,28].

In the mid-1990s, in South Baltimore, Maryland, the Air Committee of the Community Environmental Partnership (CEP) worked with EPA scientists to assess air quality [29]. The committee reviewed emission reports for more than 125 facilities and identified 175 chemicals released to, or measured in, ambient air in the CEP neighborhoods. While they could not provide risk calculations corresponding to exposure scenarios or specific to the CEP neighborhoods [29], the information was beneficial for community action because: (1) it provided an inventory of commercial, industrial, and waste treatment/disposal facilities; (2) it established a baseline for community air quality to evaluate future progress and highlight potential concerns with new sources; and (3) it provided the basis for pollution prevention and education measures for benzene, odors, and diesel truck exhaust reduction [29]. However, poor health effects and risk communication created tension and acrimony among partners. Many stakeholders disavowed the results of the study and left the partnership [29]. From the perspective of the CPS model, investigators could have obtained more spatially and community-relevant pollution and health data [30] to educate local residents, increase their environmental awareness, and enhance community capacity to develop and employ risk reduction strategies.

In the late 1990s, in the city of Spartanburg, South Carolina, the predominantly black, low-income neighborhoods of Arkwright and Forest Park were surrounded by environmental hazards, including a 40-acre fertilizer plant (a Superfund site), a public dump, a 30-acre former municipal landfill (a Superfund site), a chemical plant, textile mill, and six brownfields [31,32,33,34,35]. There were high rates of cancer, particularly bone, colon and lung, and high rates of respiratory illnesses, adult mortality, infant mortality, miscarriages and birth defects [33,34,35]. In addition, residents had poor transportation infrastructure, limited sewer and water services, lack of access to medical care, public safety issues, few economic opportunities, and declining property values [34,35]. In 1997, the ReGenesis Partnership was established by Harold Mitchell, a local resident. ReGenesis built an EJ partnership with the City of Spartanburg, Spartanburg County, EPA Region 4 Office of Environmental Justice, the South Carolina Department of Health and Environmental Control (SCDHEC), Spartanburg Housing Authority, Spartanburg County Community and Economic Development Department, local industry, and the University of South Carolina (USC) Upstate. The work of ReGenesis became the foundation for the EPA National Collaborative Problem Solving (CPS) model, which has been described by NEJAC as the way that stakeholders should collaborate to reduce and eliminate cumulative risks [18].

### 3.2. State

State agencies can also use CRA methods to better inform their decision-making process. California has been a leader in developing and implementing EJ and CRA strategies to develop policies and guide decision-making. Because of the breadth and depth of their approaches, the following section focuses on California *Policies and Regulations* and *Analytical Methods and Decision-Making*. While other states have EJ-related policies, California has been exemplary in their approaches, which represent some of the most implementable state-level strategies, and so we chose to focus on them as the standard.

#### 3.2.1. Policies and Regulations

California has invested resources in the development of new approaches to assess cumulative impacts because of EJ concerns expressed by community leaders. California passed a state EJ law and mandated an examination of how decision-making processes in its environmental programs, policies or activities could hinder EJ efforts [3,4,36]. The state implemented several legislative and policy changes to address disparities arising from cumulative environmental exposures. In 2000, the legislature named the California Environmental Protection Agency (Cal-EPA) Office of Planning and Research as the lead agency for developing EJ guidelines [4,36]. In 2003, Cal-EPA established its EJ Advisory Committee (EJAC), consisting of community members, industry, government and academia, to recommend criteria for addressing EJ gaps in programs and policies [4,32,36].

One of the first EJAC reports focused on cumulative impacts and disproportionate exposures [32], providing recommendations to develop a working definition of cumulative impacts that incorporates total pollution emissions and discharges in a geographic area; guidance on cumulative impact assessment; and criteria to implement the guidance, including changes in regulation, statutes or policy [32]. The report emphasized: (1) cumulative impact analysis should account for past, current and future emissions and discharges; (2) analyses should include quantitative, semi-quantitative, and qualitative methods; and (3) the assessment should span a geographic area large enough to encompass effects but not so large as to mask or dilute effects due to spatial averaging [3,4,32,36].

The input from EJAC helped Cal-EPA create a framework to address cumulative impacts. The framework considers: (1) exposures, public health and environmental effects; (2) all sources of emissions and discharges of pollution in a geographic area; (3) all routes of exposure; (4) routine and accidental releases; (5) sensitive populations; and (6) socioeconomic factors [4]. The input of stakeholders, government officials, and scientists led to a shift from traditional risk assessments of specific agents or pollution sources to a community- or geographic-based assessment that considers all chemical and nonchemical stressors—including land use—that may impact human health [4].

Progress has also been made to implement cumulative risk guidelines for vulnerable communities and populations. Both the California Air Resources Board (CARB) and the Bay Area Air Quality Management District (BAAQMD) have initiated projects to assess and mitigate cumulative air pollution “hot spots.” CARB established the Neighborhood Assessment Program to develop guidance on how to evaluate and address cumulative air pollution on the neighborhood scale [36]. The *Children’s Environmental Health Protection Act* was passed, which required CARB to do more to protect infants and children, including children with asthma and other susceptibilities and vulnerabilities, from air pollution exposure and impacts [36]. The BAAQMD initiated the Community Air Risk Evaluation Program to characterize cumulative air pollution risks throughout the Bay Area and take actions to reduce these risks [36]. These efforts are successful examples of how California is evaluating and addressing cumulative risks associated with air pollution at the regional and local levels.

#### 3.2.2. Analytical Methods and Decision-Making

California researchers also developed the Environmental Justice Screening Method (EJSM). EJSM is a cumulative impact mapping tool that incorporates a set of environmental, health, and social vulnerability measures in three categories: (1) hazard proximity and land use; (2) estimated air pollution exposure and health risk; and (3) social and health vulnerability [3]. EJSM facilitates evaluation of of cumulative impact patterns across neighborhoods and within regions [3]. EJSM integrates and scores multiple metrics of stressors to rank census tracts in a rigorous and transparent way, making the outputs accessible to a diverse set of stakeholders, including regulators, affected communities, industry and business [3].

An important part of the development of EJSM was the participation of a diverse set of stakeholders. These parties provided input and feedback on method development, appropriate metrics and scoring approaches [3]. CARB scientists and an external review committee provided input on methods and metrics as well. Community stakeholders and EJ advocates provided input on metrics and feedback on results during tool development. Trade-offs were made during development, including revisions to make the tool useful for community stakeholders so that they would accept it as part of regulatory guidance and environmental decision making, ensuring that the final tool was methodologically sound and user-friendly for policy makers, activists, advocacy groups, risk managers, and regulatory agencies [3].

### 3.3. Federal

Six federal laws and regulations were examined with respect to their adoption of CRA principles. Federal policies are designed to provide maximal protection at the national level, and as such are targeted toward specific compounds and/or pollution sources, and address the population as a whole. In certain instances, they consider vulnerable populations and chemical mixtures, or language on cumulative risk, but do not adopt or present a consistent approach to CRA across regulations.

#### 3.3.1. Federal Insecticide, Fungicide, and Rodenticide Act/Pesticides

The EPA, in collaboration with the states, is responsible for registering and licensing pesticides under the Federal Insecticide, Fungicide, and Rodenticide Act (FIFRA), legislated in 1947. Under its initial enactment, the FIFRA primarily focused on pesticide efficacy but later was amended by the Federal Environmental Pesticide Control Act (FEPCA) to collectively protect human health and the environment. A frequently cited CRA example is the evaluation of aggregate exposures to pesticides mandated by the Food Quality Protection Act (FQPA) of 1996, which specifically states that pesticides with a common MOA should be evaluated for their human health risks [27], such as for organophosphorus (OP) pesticides with an MOA of acetyl cholinesterase [AChE] inhibition [27,37,38]. In the case of pyrethroid pesticides (type I and type II), additive health effects cannot be assumed because they do not have a unified MOA [27,37,38]. One criterion for registering a pesticide under FIFRA is that “it will perform its intended function without unreasonable adverse effects on human health and the environment” (FIFRA Sn. 3).

#### 3.3.2. Clean Water Act

In 1977, the Clean Water Act (CWA) replaced the Federal Pollution Control Act of 1948, initially created to address water pollution. CWA provided a comprehensive approach to controlling water pollution by: (1) establishing a framework for regulating pollutant discharges into U.S. waters; (2) providing the EPA with the authority to implement water pollution control programs by setting wastewater standards for industry; (3) using existing water quality standards to set additional criteria for controlling contaminants in surface water; (4) creating legal ramifications for persons who discharge pollutants from a point source into a water system unless permitted under specified provisions; (5) funding the construction of sewage plants under the construction grants program; and (6) incorporating planning to address water pollution problems caused by nonpoint source pollution. The EPA partnered with federal, state, and tribal organizations to assure compliance and enforcement of the CWA [39]. The CWA calls for standards “adequate to protect public health and the environment from any reasonably anticipated adverse effects” (CWA Sn. 405 (d)(2)(D)).

#### 3.3.3. Safe Drinking Water Act

The Safe Drinking Water Act (SDWA) was passed by Congress in 1974 to protect public drinking water from naturally occurring and man-made contaminants. Amendments in 1986 and 1996 expanded legislation to include rivers, lakes, reservoirs, springs, and groundwater wells [40]. The EPA Office of Drinking Water (ODW) combines chemical risks from ingestion of drinking water by aggregating and summing chemicals with common target-organ effects [8]. In situations where it has been necessary to determine health risks associated with a mixture of disinfection byproducts (DBPs) in publicly regulated potable-water supplies, the ODW used guidance provided in the Guidelines for the Health Risk Assessment of Chemical Mixtures and Supplemental Guidance as well as DBP studies [41,42]. The National Primary Drinking Water Regulation (NPDWR) standards limit contaminant levels in the environment that can adversely affect health. These levels are further specified under the EPA Maximum Contaminant Level Goals (MCLGs). An extensive review of health effects studies, as well as special considerations for vulnerable subpopulations (*i.e.*, infants, children, elderly, and persons with compromised immune systems), are evaluated to determine appropriate MCLG guidelines.

#### 3.3.4. Comprehensive Environmental Response, Compensation, and Liability Act (Superfund)

President Carter and the U.S. Congress enacted the Comprehensive Environmental Response, Compensation, and Liability Act (CERCLA) in 1980 to mitigate the burden of hazardous waste sites [43]. CERCLA established requirements for closed and abandoned sites, allowed persons to be held legally accountable for releases of hazardous wastes, and created a billion-dollar trust fund (hence the name Superfund) when no responsible party can be identified to remediate a site. The 1986 Superfund Amendments and Reauthorization Act (SARA) [44] made the following changes: (1) focus on permanent solutions and innovative technologies; (2) consider standards of other state and federal environmental policies; (3) establish enforcement authorities and settlement tools; (4) increase state involvement; (5) focus on human health problems; (6) increase community participation; and (7) expand trust fund resources to $8.5 billion [44]; however, the trust fund is no longer active at this time. The Risk Assessment Guidance for Superfund (RAGS) represents a baseline human health risk assessment (http://www.epa.gov/oswer/riskassessment/ragsa/) that occurs after a site has been assigned to the National Priorities List (NPL), during the “remedial investigation.” RAGS uses an additive framework for pollutants with a common MOA [45].

#### 3.3.5. Clean Air Act

The Clean Air Act (CAA) was enacted to regulate air emissions and protect health. The original CAA of 1963 was motivated by events in Pennsylvania and London, during which people became ill or died from lingering smog. The CAA provided funding to research air pollution and identify solutions. In 1970, the EPA implemented an improved version of the CAA, and was given the responsibility of enforcing air quality guidelines using the most cost-effective approaches [46]. The final 1990 amendment established the National Ambient Air Quality Standards (NAAQS) for specific pollutants. Primary and secondary air quality standards were established for the following pollutants: carbon monoxide, lead, nitrogen dioxide, particulate matter (PM_10_ and PM_2.5_) ozone, and sulfur dioxide. Primary standards set limits to protect vulnerable populations, particularly children, the elderly, and asthmatics. Secondary standards set limits related to reduced visibility and damage to animals, vegetation, and buildings.

#### 3.3.6. National Environmental Policy Act

The National Environmental Policy Act (NEPA) of 1969 requires an environmental assessment for projects undertaken, funded, or permitted by public agencies to address potentially adverse effects to land, air, water, minerals, plants and animals, among others [4,47,48]. The Council on Environmental Quality (CEQ) was created to ensure proper implementation of NEPA. CEQ regulations require that agencies consider “the direct, indirect, and cumulative effects” of the proposed action and alternatives, and define health as one of the effects to include [49]. Beneficial effects may also be included [48,49]. Agencies are further directed to consider how “economic or social and natural or physical environmental effects are interrelated” [48]. The regulations and available guidance, however, do not identify specific methods to analyze health or other effects in the environmental impact statement (EIS) [47,49]. Instead, NEPA requires that agencies “utilize a systematic, interdisciplinary approach which will ensure the integrated use of the natural and social sciences and the environmental design arts” [48,49].

## 4. Results

Results are divided into the three phases of a CRA: (1) planning, scoping, and problem formulation, (2) risk analysis, and (3) risk characterization and interpretation [1]. A synopsis is provided for community, state, and federal applications as they pertain to the elements in the different phases (Figure 1). Even though these CRA elements represent a framework [1], as opposed to an established methodology, they nonetheless provide a valuable perspective on the relative differences between the different types of applications, which are then used to develop recommendations for a consistent approach to CRA in the Discussion.

### 4.1. CRA Planning, Scoping, and Problem Formulation

Planning, scoping, and problem formulation (Table 2) represent Phase 1 of the CRA framework [1]. The first column of Table 2 is identical to the primary sub-points that should be included in these sections (Figure 1). Planning and scoping includes defining the purpose, scope, participants, approach, resources, and past experiences. Problem formulation entails development of a conceptual model and analysis plan, and findings could be used to further inform planning and scoping. The discussion of possible outcomes occurs early and also informs planning, scoping, and problem formulation.

### 4.2. CRA Risk Analysis

Phase 2 of a CRA addresses risk analysis (Table 3), including the integration of exposure, hazard, and dose-response information, and—in order of increasing complexity—single stressor information, multiple stressor information, and measures and metrics to quantify multiple stressors. The first column of Table 3 is identical to the primary sub-points that should be included in these sections (Figure 1).

### 4.3. CRA Risk Characterization, Interpretation, and Management

Phase 3 of a CRA includes risk characterization, interpretation, and management (Table 4). While risk management is not explicitly included in the Framework for Cumulative Risk Assessment [1], it has been recommended to include it early in the CRA and during the interpretation stage because of the importance of prioritizing solutions based on stressor magnitude and the feasibility of addressing them [50].

**Table 2:** Synopsis of Planning, Scoping, and Problem Formulation elements for Phase 1 of a CRA for Community, State, and Federal applications.

Planning, Scoping, Problem Formulation Elements	Community	State	Federal
Planning and Scoping			
Purpose	Improve community health	Allocate/distribute resources to protect residents from environmental harm	Maximal protection of population as a whole; improve conditions at local levels
Scope	Neighborhood area(s); current conditions; historical exposures; future projections; population-based; precautionary	Geo-political boundaries; community scales; urban, suburban, and rural scales; pollution regulation; land maintenance; infrastructure; transportation; social, environmental, and economic considerations (*i.e.*, sustainability) for planning	Sector and chemical-driven protection; cost-effective solutions (e.g., CAA); principally reactive in origin (e.g., CERCLA); predictive as well (e.g., MOA grouping in FIFRA); agencies adopting local-scale principals (e.g., Superfund RAGS)
Participants	Local residents (e.g., Chester, PA); agencies (e.g., South Baltimore); academics and health departments (e.g., Spartanburg, SC)	Representative councils (e.g., EJAC); stakeholder input (e.g., EJSM) Locally-driven initiatives (e.g., BAAQMD)	Expert solicitation (e.g., SDWA); local considerations (e.g., NEPA) Multi-stakeholder involvement (e.g., SARA)
Approach	Participatory	Interactive	Reflective
Resources	Human; financial; technical; political	Policy-driven allocation	Distributed across agencies
Past Experiences	Anecdotal; perceived risk; historical perspectives on exposure; local knowledge of health and environment	Multi-faceted (social, environmental, economic) perspective on impacts and decision-making	Historical records and lessons learned domestically and abroad
Problem Formulation			
Conceptual Model Sources Stressors Pathways/Routes Receptors Endpoints	Network of partners and collaborators; linkages between stressors and solutions	Environmental and health predictions with sustainability considerations	Establish baseline and modifications to exposure/response due to multiple stressors
Analysis Plan Methods Models Data Gaps Uncertainties	Data informs decision-making and defense of risk analysis, characterization, and management options	Data identifies populations of interest and informs allocation of resources	Quantitative approaches with modes of action (MOAs) and maximum contaminant level goals (MCLGs) inform standards
Discussion of Possible Outcomes	Develop and adopt local initiatives/policies implemented by residents or government; work with intentionality	Achieve sustainable use of available social, environmental, and economic resources	Protect human health and environment across country, while maintaining global perspective

**Table 3:** Synopsis of Risk Analysis elements for Phase 2 of a CRA for Community, State, and Federal applications.

Risk Analysis Elements	Community	State	Federal
Integration of Exposure, Hazard, and Dose-Response Information Considering:			
Time Related Aspects Vulnerability Subpopulations with Special Features	Analytic-deliberative methods linking decision analysis and risk assessment	Indexes of cumulate risk (e.g., EJSM); indicators and surrogates as proxies for exposure and risk	Providing protective standards for human health based on best available toxicity and exposure relationships
Single Stressor Information			
Toxicological Independence Toxicological Similarity	Chemical mixtures from multiple sources; non-chemical stressors and other exposure/response modifiers	Implement regulations with permitting, oversight, management, and public initiatives or programs	Regulations and mixtures limited to chemically similar stressors (e.g., pesticides); also site- or source-specific (e.g., Superfund, CAA)
Multiple Stressor Information			
Stressor Interactions Joint Chemical Toxicity	Relative risk of stressors for prioritization of actions; determination of environmental impacts on health	Consideration of social determinants of health	Determination of environmental impacts on health
Measures and Metrics			
Decision Indices Probabilistic Approaches Qualitative Approaches Common Metric Biomarkers	Data collection and consolidation informs decision making and supports local initiatives	Consolidation of multiple aspects of sustainability addresses state-level decisions about resources and priorities	Impact-driven assessments of environmental stressors on human health and ecosystems

**Table 4:** Synopsis of Risk Characterization and Interpretation elements for Phase 3 of a CRA for Community, State, and Federal applications.

Risk Characterization and Interpretation Elements	Community	State	Federal
Risk Description			
Central Tendency and High-End Individual Risk Population Risk Risk to Important Subpopulations	Multiroute chemical risk assessments; poverty and race/ethnicity considerations; children and elderly; mortality/morbidity clusters	Sensitive/vulnerable population groups; socioeconomic factors; multiple emissions and discharges; current and future conditions	Standards to protect most sensitive populations (e.g., SDWA); aggregate exposure regulations (e.g. FQPA); reasonably anticipated adverse effects (e.g., CWA Sn. 405); primary standards to protect children, elderly, asthmatics
Uncertainty Analysis			
Being Explicit about Uncertainty Uncertainty and Variability Uncertainty and Risk Addition Sensitivity Analysis	GIS-based analyses; local health and emissions records; deviations from baseline or more ideal conditions; proxies for exposure; measurements and sensors increase certainty	Indicators or surrogates of exposure, such as hazard proximity and air pollution exposure estimates; resolution suitable for targeting and implementation of policy	Economic, social, and environmental conditions are interrelated, producing direct, indirect and cumulative effects
Information Provided by CRA	Stressor, asset, and resource identification; absolute or relative ranking; remediation options	Identification of at-risk individuals or populations; weighting of risk based socio-economic, health, and environmental conditions	Systematic, interdisciplinary approaches; integration of natural, social, and environmental sciences and designs
Using the Results of CRA	Solution-oriented, data-supported, value-driven decision-making	Implementation of exposure and risk reduction actions; source attribution; protective standards for land use or other policies	Dose addition with relative potency and toxic equivalency factors or to develop a hazard index; stakeholder feedback and participation to inform research and development that supports local efforts

## 5. Discussion

### 5.1. Similarities and Differences in Community, State, and Federal CRA Phases

Not all the case study examples were intended to represent a complete CRA process, especially in regards to the federal laws and regulations. However, a comparison of the different elements of a CRA framework helps to identify research gaps and integration opportunities, and informs development of a consistent procedural methodology across community, state, and federal applications.

Phase 1 of a CRA differs in several ways across the three scales. In general, the purpose of all groups is to protect health, yet subtle differences in even this first element of this phase are still obvious: improving health is often the sole concern of communities; states consider available resources and allocation measures to help develop suitable programs; and federal approaches attempt to provide maximal protection to known stressors for the majority of the population (sometimes at the expense of multi-stressor or vulnerable population considerations). The scope and participants can vary, but typically, communities focus on neighborhood applications that are driven by resident participation and engagement; states adopt feedback from stakeholder and expert partnerships to develop policies and initiatives; and federal approaches solicit expert advice and stakeholder feedback to help develop national policies. The approaches reflect this: communities are highly participatory, states are interactive, and federal policies are reflective in that they respond to proven health issues. Community must consider human, financial, technical (e.g., data analysis or exposure models), and political resources; states develop policies and initiatives to allocate resources; and federal approaches to CRA must often cross several agencies to account for all stressors and issues. Past experiences in communities can draw from anecdotal evidence and local knowledge; states consider the interplay between social, environmental, and economic challenges; and federal approaches draw on historical records and international examples.

In terms of problem formulation, community-based conceptual models explicitly include potential solutions and risk management options and how they relate to stressors; states rely on future projections and sustainability when examining stressor interactions; and federal approaches seek to establish baseline conditions and quantify exposure/response modifiers that might increase the likelihood or consequence of a stressor exposure. The analysis plan in community settings informs decision-making; at the state level, it identifies at-risk populations and informs resource allocation; and for federal applications, it focuses on quantification of toxicological impacts and evaluating uncertainties. The discussion of possible outcomes is most relevant at the community level, since the purpose of the ensuing risk analysis is usually to isolate feasible corrective actions; states seek to achieve long-term sustainable outcomes; and federal approaches attempt to provide environmental health protection across the country, while maintaining a global perspective on lessons learned and approaches.

Phase 2 of a CRA refers to risk analysis. For conventional risk assessment, this relates to the exposure and dose-response assessment portions, attempting to quantify risk impacts. For community settings, decision-making (informed by decision analysis sciences) and risk assessment are both important. Often, for communities, the primary interest is on identifying multiple stressors with a common impact (e.g., air pollution and fugitive dust on asthma), or on comparing the relative risk of stressors based on absolute risk and community values. States such as California have adopted indicators or surrogates of exposure, such as proximity to hazardous sources, sensitive land use (e.g., daycare centers), and poverty to develop a consolidated index of cumulative impacts; while often representative, it can be difficult to capture the uncertainty of surrogates in estimating exposure. Federal approaches to risk analysis are strongly focused on like-chemical assessments, such as pesticides, and rely on quantitative measures of toxicity to establish regulatory standards; they rarely account for multiple stressors (exceptions being Superfund and NEPA) or mixtures, even in overarching mandates like CAA or CWA.

Phase 3 addresses risk characterization and interpretation. Because of the solution-oriented recommendations set forth by the National Research Council [50], the development of risk management options can also be implemented in this phase. Two of the main topics to consider include the description of risk, especially as it pertains to sensitive subpopulations, and an uncertainty analysis that explains explicitly the limitations of the risk analysis. For communities, the risk description often encapsulates multi-stressor analyses, non-chemical and vulnerability considerations, and health incidence clusters. The uncertainty characterization could be narrative based on the level of quantification used during risk analysis, but be supplemented by analytical tools like GIS or citizen science measurements. For states, uncertainty can be characterized based on the impact to sensitive subpopulations, inclusion of socio-economic factors, and the probability of future projections. The use of indicators also introduces uncertainty, and may only provide a general identification of cumulative impacts instead of an accurate risk estimate due to, for example, personal exposure levels. Federal regulations include consideration of sensitive subpopulations and reasonably anticipated adverse effects, which can be interpreted based on the application. While federal regulations are often targeted toward specific pollutants or sectors, they acknowledge the interrelated and cumulative effects of economic, social, and environmental conditions. The information provided by a CRA helps communities to identify and rank stressors, and prioritize solutions; the results help to inform decision-making by residents and local authorities. States use CRA information to identify at-risk populations, weighting risk based on environment and health information as well as socio-economic and related conditions. The goal of state-level information is often used to implement exposure and risk reduction initiatives, identify primary stressor sources, and allocate resources. Federal-level information adopts systematic, interdisciplinary approaches to integrate natural, social, and environmental sciences. This information helps to develop dose addition strategies that can be used to set a baseline of exposure/response to stressors with known outcomes; in addition, this helps to identify exposure/response modifiers that might increase risk and adverse impacts.

### 5.2. Research Gaps and Recommendations for a Consistent CRA Process

As researchers adapt and apply methods for CRA, then the identification, prioritization, and mitigation of stressors will begin to address multiple environmental health concerns not only simultaneously, but with a range of solutions that include social, environmental, and economic approaches. Health impact assessment (HIA) is one of the newer approaches that focuses on a given set of health impacts, such as cancer clusters or childhood asthma attacks, and then explores the range of contributing stressors and stressor sources. However, even with HIA, data collection and analysis, risk ranking, and solution prioritization are largely left to the user, and no gold standard has yet been established [51]. CRA should provide structured and scientifically sound guidance for each step of the assessment process, from forming partnerships and defining objectives, to risk ranking and solution prioritization. To that end, CRA and HIA can both benefit from additional research to determine the most effective and efficient methods.

A consistent CRA procedural methodology is not intended to replace the tools that communities, states, and federal authorities need in order to derive actions or set mandates. Rather, it is intended to provide a common ground between entities that each can recognize as a robust and transparent assessment process, backed by science and intended to inform decision-making. The level of quantification and objectives will vary between applications, but the process would reflect the most important components of a CRA and offer a step-by-step process for achieving goals.

We investigated similarities and differences in risk assessment approaches at the community, state, and federal levels, and isolated the most important aspects that would fulfill the requirements of a CRA. Some of the most important aspects include the formation of a collaborative partnership and the open discussion of goals and objectives; the collection and analysis of appropriate data; the subsequent ranking of disparate multiple stressors; and the prioritization of solutions based on available resources and feasibility. Whether a CRA is initiated by a community, state, or federal group, these components should be incorporated; otherwise, the terms “cumulative” and “assessment” are not well-represented. HIAs can also benefit from a more structured approach, and the development of scientifically sound quantification approaches that can be developed by researchers, policy makers, community leaders, and impacted individuals. One other research gap is to bring together these people in order to develop appropriate methodologies together, in order to avoid independent development of methods that are not accepted by others.

1. Define Purpose—the main goal of the CRA around which analysis, characterization, and management are implemented.
2. Define Objectives—objectives of each group and individual, for transparency and in support of the purpose; to extent possible, these should be achievable and measureable.
3. Engage Partnership—determine the core personnel responsible for conducting the CRA and seeing it through to completion, and identify stakeholders, experts, agencies and others to invite, either as ongoing partners or as consultants on specific topics or for a limited timeframe.
4. Define Roles and Responsibilities—clearly articulate the role of each partner in conducting the CRA, and the specific responsibilities for which they will be held accountable.
5. Determine Scope—temporal (e.g., historical, current, or future conditions), spatial (e.g., neighborhood, state, or national), receptors (e.g., defined community or sensitive subpopulations), and the level of information/quantification needed to make a decision (e.g., qualitative informational evidence, semi-quantitative indicators or surrogates, or quantitative absolute toxicological risk estimates).
6. Identify Stressors and Assets—create a broad list of the primary issues of concern, and identify any related and possibly synergistic or antagonistic stressors or assets, respectively (assets are benefits to a CRA, either by reducing a risk or building capacity to address them); a conceptual model is often useful, but not necessary for this step.
7. Rank Stressors—implement a meaningful risk ranking methodology; because of the analytic-deliberative nature of CRAs, it is advisable to develop methods that can consolidate multiple stressors into a single risk estimate, as well as to develop methods to assess the relative risk between stressors, which can be accomplished by integrating risk assessment and decision analysis into a common framework.
8. Prioritize Solutions—use results of the stressor ranking to develop and prioritize solutions, based on the ability of risk-reduction efforts to address multiple stressors, high-ranking stressors, or on the feasibility of implementation (*i.e.*, taking actions against risks that can easily be targeted with available resources in order to build capacity and remediate obvious stressors first).
9. Summarize Analysis Plan—based on information collected and analytic-deliberative outcomes, detail the precise approach required to perform the CRA.
10. Evaluate Results of Risk Reduction Actions—after implementing solutions and risk management options, develop measures of success to track effectiveness and adapt planning.

Each of these steps should be documented and the analysis procedures open for interpretation and scrutiny (*i.e.*, transparent). Even though many projects, initiatives, and programs inherently include these steps to some degree, a consistent approach would develop best practices for each, to explicitly address them and advise how they can be achieved. Templates, recommended approaches, and best practices could be developed and provided for each step to promote consistency and acceptability of results.

## 6. Conclusions

Community, state, and federal approaches to CRA (or general risk assessment) share the common goal of protecting human health and the environment; however, their approaches are largely determined by their goals—communities seek to improve local, neighborhood-level health; states need to allocate resources and develop appropriate local-scale, targeted initiatives; and federal applications seek to maximally protect health for the population as a whole, with standards developed to protect the most sensitive subpopulations.

Probably the most deficient CRA element relates to risk analysis—the quantification of multiple stressors. Mixtures toxicity is a challenge unto itself, grouping chemicals based on MOAs or toxicity pathways (*i.e.*, the biological malfunction that they cause), so characterizing disparate stressors without a common endpoint proves exceptionally challenging. Until the science has advanced enough to analyze cumulative impacts as an absolute measure of risk, an alternative is to develop relative risk ranking procedures to compare disparate stressors based on exposure or risk surrogates or other data-driven estimates of risk.

While risk assessment has often been relegated to determining the odds of a stressor impact as an end unto itself, CRA includes consideration of risk management options and the prioritization of solutions as an integral and necessary part of the assessment. Solution possibilities should be considered early in the assessment, and then further prioritized based on the findings of the risk ranking. To this end, a CRA not only analyzes multiple stressors, but devises solutions for remediating them.

In all, communities, states, and federal agencies have begun to develop methods for conducting CRAs, but it has yet to be well-established as to which methods are most acceptable across entities, and the extent to which they can be used to inform decision-making. In order to advance CRA research and development, we recommend that a consistent approach be developed that relates to the most cross-cutting and relevant aspects of the assessment. For each step of the approach, best practices and recommended approaches can be provided to promote communication and acceptance of results across community, state, and federal levels.

HIA has been used impressively by mostly academic and policy researchers who knew what types of information they needed, where to collect it, and how to compile it into broad reports on environment and health [51]. However, HIA, like CRA, has no commonly-agreed upon approach either, and therein lies some of the difficulty. Communities who would like to use CRA or HIA as a tool are largely not represented in the literature because they are specifically the ones who do not have the capacity to carry out those studies, especially with the lack of specific instructions on how to do them. We would argue that it is time to move beyond conceptual approaches and into the realm of standardized consistency, hence the 10 steps described in the paper. Each step should be documented for a CRA, and each step should provide a recommended approach or approaches that can easily be adopted, either by providing templates or a computerized interface, for example, and based on the best available scientific approaches.

Because of the nature of the research presented here, we can only present our best interpretation of the steps or components that would be essential to include in any cumulative assessment—one that includes multiple stressors, participants, perspectives, objectives, and approaches to solutions. While admittedly subjective, the examples and discussions support these conclusions; the absence of one or more of these steps would compromise the integrity of CRA and be left in the realm of yet another project-specific assessment with an approach that is difficult, if not impossible, to transfer to other places or applications.

## 7. Disclaimer

This article been subject to review by the EPA and approved for publication. Although this work was performed as research for the U.S. Environmental Protection Agency, it does not necessarily represent endorsement of official Agency policies.
